# Exploring the Protective Effects of Traditional Antidiabetic Medications and Novel Antihyperglycemic Agents in Diabetic Rodent Models

**DOI:** 10.3390/ph18050670

**Published:** 2025-05-01

**Authors:** Cosmin Gabriel Tartau, Ianis Kevyn Stefan Boboc, Liliana Mititelu-Tartau, Maria Bogdan, Beatrice Rozalina Buca, Liliana Lacramioara Pavel, Cornelia Amalinei

**Affiliations:** 1 Department of Morphofunctional Sciences I, Faculty of Medicine, ‘Grigore T. Popa’ University of Medicine and Pharmacy, 700115 Iasi, Romania; cosmin.tartau@gmail.com (C.G.T.); cornelia.amalinei@umfiasi.ro (C.A.); 2 Department of Pharmacology, Faculty of Pharmacy, University of Medicine and Pharmacy of Craiova, 200349 Craiova, Romania; bogdanfmaria81@yahoo.com; 3 Department of Pharmacology, Faculty of Medicine, ‘Grigore T. Popa’ University of Medicine and Pharmacy, 700115 Iasi, Romania; lylytartau@yahoo.com (L.M.-T.); beatriceroza@yahoo.com (B.R.B.); 4 Department of Morphological and Functional Sciences, Faculty of Medicine and Pharmacy, ‘Dunarea de Jos’ University, 800010 Galati, Romania; doctorpavel2012@yahoo.com

**Keywords:** diabetes mellitus, histopathology, antihyperglycemic agents, metformin, tirzepatide

## Abstract

Type 2 Diabetes (T2D) is a complex metabolic disorder that affects multiple organs, leading to severe complications in the pancreas, kidneys, liver, and heart. Prolonged hyperglycemia, along with oxidative stress and chronic inflammation, plays a crucial role in accelerating tissue damage, significantly increasing the risk of diabetic complications such as nephropathy, hepatopathy, and cardiovascular disease. This review evaluates the protective effects of various antidiabetic treatments on organ tissues affected by T2D, based on findings from experimental animal models. Metformin, a first-line antidiabetic agent, has been widely recognized for its ability to reduce inflammation and oxidative stress, thereby mitigating diabetes-induced organ damage. Its protective role extends beyond glucose regulation, offering benefits such as improved mitochondrial function and reduced fibrosis in affected tissues. In addition to traditional therapies, new classes of antidiabetic drugs, including sodium-glucose co-transporter-2 inhibitors and glucagon-like peptide-1 (GLP-1) receptor agonists not only improve glycemic control but also exhibit nephroprotective and cardioprotective properties by reducing glomerular hyperfiltration, oxidative stress, and inflammation. Similarly, GLP-1 receptor agonists have been associated with reduced hepatic steatosis and enhanced cardiovascular function. Preclinical studies suggest that tirzepatide, a dual GLP-1/gastric inhibitory polypeptide receptor agonist may offer superior metabolic benefits compared to conventional GLP-1 agonists by improving β-cell function, enhancing insulin sensitivity, and reducing fatty liver progression. Despite promising preclinical results, differences between animal models and human physiology pose a challenge. Further clinical research is needed to confirm these effects and refine treatment strategies. Future T2D management aims to go beyond glycemic control, emphasizing organ protection and long-term disease prevention.

## 1. Introduction

Type 2 Diabetes (T2D) is the most common form of the disease, primarily affecting adults and resulting from insulin resistance or inadequate insulin production [1]. Over the past three decades, its global prevalence has risen significantly [2]. Type 1 Diabetes (T1D), formerly known as juvenile or insulin-dependent diabetes mellitus (DM), is a chronic condition in which the pancreas produces little to no insulin [3].

Although DM is primarily considered a metabolic disorder, it has far-reaching effects on various organs, including the heart, kidneys, liver, and peripheral nerves, impacting overall health. Chronic hyperglycemia is a key driver of structural and functional damage in these organs, with complex underlying mechanisms such as advanced glycation end-products, oxidative stress, chronic inflammation, and metabolic dysfunction [4,5].

T2D is characterized by increased insulin resistance combined with impaired insulin secretion, leading to excessive glucose accumulation in the bloodstream and subsequent organ damage [6]. Chronic hyperglycemia contributes to tissue injury through multiple pathways, including protein glycation, oxidative stress, and inflammatory activation [7]. The excessive glycation of proteins promotes the formation of advanced glycation end-products, which bind to specific receptors, triggering inflammatory cascades and oxidative stress [8,9]. Hyperglycemia also generates free radicals, causing cellular and tissue damage that leads to inflammation and functional impairment of organs. This process plays a central role in the development of long-term diabetic complications, such as retinopathy, nephropathy, and neuropathy [10].

Chronic inflammation is a hallmark of DM, mediated by inflammatory cytokines and other molecular signals that contribute to organ damage, particularly in blood vessels and kidneys [11,12]. T2D is a progressive disease, with insulin resistance often developing before the onset of hyperglycemia. As long as pancreatic beta cells can compensate by producing sufficient insulin, blood glucose levels remain relatively stable. However, T2D manifests when beta-cell function declines, leading to insufficient insulin secretion and the inability to maintain normoglycemia [7].

The development of T2D is influenced by a combination of environmental and genetic factors [13]. Various physiological abnormalities contribute to the disruption of glucose metabolism, including increased insulin resistance in skeletal muscles, the liver, and adipose tissue, as well as impaired insulin secretion by pancreatic beta cells [14]. Additional abnormalities include elevated glucagon levels, basal hepatic glucose production, lipolysis, and high levels of free fatty acids, without a decrease in these levels postprandially. There is also suppression of intestinal incretins, such as GLP-1 and GIP postprandially, associated with beta-cell resistance to the stimulatory effects of GLP-1 and GIP, leading to pancreatic beta-cell failure [12]. Increased renal glucose reabsorption via SGLT-2, neurochemical changes promoting weight gain (including leptin resistance, amylin resistance, and reduced brain dopamine), chronic inflammation, and vascular tone impairment represent additional factors contributing to hyperglycemia [15].

Chronic effects of DM result from various disruptions in metabolic pathways, contributing significantly to the morbidity and mortality associated with the disease [16]. Traditionally, these effects have been classified as microvascular or macrovascular [17]. However, it is now recognized that they can be categorized as vascular, parenchymal, or hybrid in nature, involving both vascular and parenchymal components. This distinction arises because the clinical, biochemical, and histological profiles of these conditions differ depending on whether the damage is confined to vascular tissues, primarily affects parenchymal organs, or it involves both components [18].

In recent years, the focus of research has shifted from solely identifying risk factors to understanding the interaction between both risk and protective factors in the development of DM-related damage. While risk factors remain crucial in the progression of DM’s adverse effects, emerging studies suggest that protective factors also play a key role in modulating the severity and development of these issues [13,19]. These protective mechanisms may explain the variability in the severity of organ damage and the absence of pathological changes in certain tissues [20].

Vascular complications predominantly affect various components of the cardiovascular system, including arteries, renal glomeruli, the myocardium, and blood vessels in the central nervous system [21]. On the other hand, parenchymal tissue complications are involved in the development of neoplasms, Alzheimer’s disease, and osteoarthritis [22]. In the case of osteoarthritis, the presence of DM can impair bone remodeling, increase synovial inflammation, promote chondrocyte apoptosis, and alter the extracellular matrix structure in both bone and cartilage. Additionally, many chronic complications of DM arise from associated abnormalities in vascular tissues and parenchymal structures [18].

In the past, the treatment of T2D was primarily focused on reducing blood glucose levels. However, over time, with accumulated clinical experience, therapeutic strategies have shifted toward addressing the pathophysiological disruptions caused by the disease, particularly the attenuation of cardiorenal risk, and even toward organ protection [23]. These goals can be achieved by using combinations of agents that target different pathophysiological disturbances or by employing modern antihyperglycemic agents with multiple mechanisms of action. Due to the multifaceted nature of this metabolic disease, the use of multiple drug therapies is necessary for effective glucose control and reducing the risk of vital organ damage [24,25].

Despite significant advances in pharmacological treatments and continuous updates to DM management guidelines, the rate of achieving therapeutic goals remains below expectations. Multiple classes of medications are approved for treating T2D: biguanides (e.g., metformin), sulfonylureas, thiazolidinediones, dipeptidyl peptidase 4 inhibitors, SGLT-2 inhibitors (empagliflozin, ertugliflozin, dapagliflozin, canagliflozin), GLP-1 analogs (semaglutide, liraglutide), dual GLP-1 receptor agonist and GIP analog (tirzepatide), retatrutide (a triple agonist of GIP, GLP-1, and glucagon receptors), insulins, α-glucosidase inhibitors, dopaminergic antagonists, bile acid sequestrants, meglitinides, and amylin analogs [24,26,27,28,29,30]. Some of these antidiabetic drugs have garnered particular interest due to their potential protective roles on the function and structure of various organs, especially the heart, kidneys, liver, and brain.

The goal of this review is to highlight the impact of innovative antihyperglycemic treatments on the function and structure of vital organs in animals with experimentally induced T2D, focusing on next-generation agents, such as SGLT-2 inhibitors and GLP-1 agonists, and their effects on organ damage associated with DM.

## 2. Structural Organ Changes in T2D

**Figure 1:**
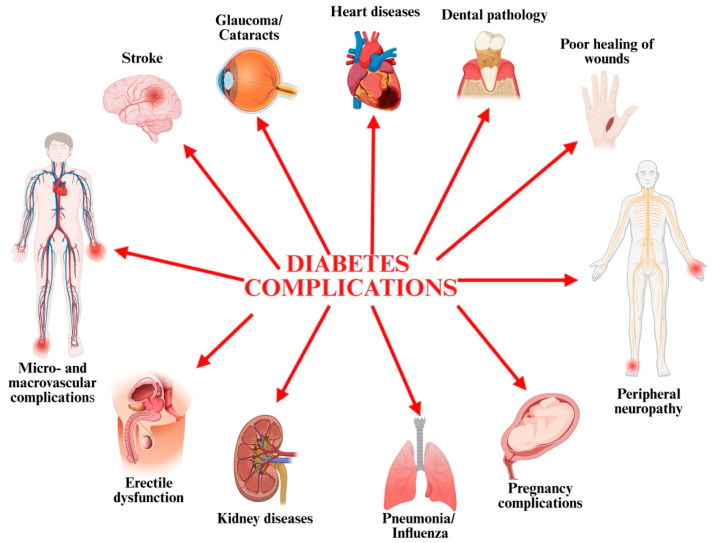
The complications of DM (created with BioRender.com).

Hyperglycemia results in metabolic dysfunctions of carbohydrates, fats, and proteins, leading to the disruption of function and structural damage to multiple organs in the body (Figure 1) [11,20,31].

Ocular complications lead to retinopathy, which can progress to blindness. Renal complications can cause nephropathy, potentially progressing to kidney failure [32,33]. Nerve-related complications result in neuropathy, which can be both autonomic and/or peripheral. Cardiovascular, gastrointestinal, and genitourinary dysfunctions are characteristic manifestations of autonomic neuropathy, while long-term peripheral neuropathy is often associated with foot infections, which may even require amputations [17,34].

DM is accompanied by profound histological changes in various organs, largely resulting from prolonged hyperglycemia, which causes damage through complex mechanisms such as protein glycation, oxidative stress, and activation of inflammatory pathways [9].

In the pancreas, the islets of Langerhans undergo notable changes, especially in T2D, where there is a significant reduction in beta-cell mass due to apoptosis and amyloid deposits, leading to the replacement of functional beta cells and contributing to insulin deficiency [6].

The kidneys, a key target in DM, experience characteristic structural and functional changes in diabetic nephropathy, including thickening of the glomerular basement membrane, mesangial expansion, and glomerulosclerosis, which ultimately lead to proteinuria and progressive kidney failure [35].

The cardiovascular system is also profoundly affected. DM accelerates the progression of atherosclerosis, leading to coronary artery disease and myocardial infarction. The aorta and other large arteries exhibit thickening of the intima and increased lipid accumulation, while smaller arteries and arterioles show hyaline arteriolosclerosis [36]. These vascular changes are further exacerbated by diabetic cardiomyopathy, where myocardial fibrosis and microvascular dysfunction contribute to the development of heart failure [37].

The liver also shows signs of non-alcoholic fatty liver disease, a common comorbidity in DM, and histologically, it may present with steatosis, ballooning degeneration of hepatocytes, and fibrosis, potentially progressing to cirrhosis [38]. In the brain, several changes may occur, including neuronal loss, particularly in regions like the hippocampus (which plays a role in memory and cognitive function), microvascular lesions with reduced cerebral blood flow, white matter abnormalities, and microglial activation [39]. Overall, the histological changes observed in various organs within the context of DM highlight the systemic nature of the disease and emphasize the importance of glycemic control to prevent or reduce these extensive tissue injuries.

This complex disease presents a wide range of clinical manifestations and significant individual differences in treatment response. Standard treatment regimens may not adequately meet the individualized needs and unique characteristics of each patient, often leading to suboptimal results and difficulties in achieving optimal glycemic control [40]. In light of these challenges, personalized medicine has emerged as a promising solution, adapting treatment plans and interventions according to the patient’s individual characteristics for improved treatment efficacy and enhanced quality of life [41,42].

## 3. Metformin—Traditional Antidiabetic Agent

Metformin (MET) is an antihyperglycemic medication from the biguanide class, widely used in the treatment of T2D. Its primary mechanism of action involves reducing hepatic glucose production by inhibiting gluconeogenesis and glycogenolysis [43]. MET works by activating AMP-activated protein kinase, which subsequently decreases the expression of enzymes involved in glucose synthesis [44]. Additionally, it enhances insulin sensitivity by increasing glucose uptake and utilization in skeletal muscles and adipose tissue [45]. Furthermore, MET slows down intestinal glucose absorption, contributing to a reduction in postprandial hyperglycemia [46]. Another beneficial effect of MET is its impact on the gut microbiota and fatty acid metabolism, which may play a role in its effects on body weight and metabolic profile [47]. Unlike other antidiabetic agents, MET does not stimulate insulin secretion, thereby reducing the risk of hypoglycemia [24].

Beyond its use in DM, MET is being studied for its effects in polycystic ovary syndrome, DM prevention in prediabetic patients, and its potential benefits in metabolic diseases and aging [48,49]. Due to its effectiveness and favorable safety profile, MET remains the cornerstone in the treatment of T2D, often used alone or in combination with other antidiabetic medications for optimal glycemic control [50,51].

Extensive evidence from animal models of DM has demonstrated that MET exerts significant protective effects on the retina through multiple mechanisms that extend beyond glycemic control. In streptozotocin (STZ)-induced diabetic rats and mice, MET treatment leads to the activation of AMP-activated protein kinase (AMPK), a key regulator of energy balance and cellular stress responses [52]. AMPK activation results in the inhibition of the nuclear factor-kappa B (NF-κB) signaling pathway, leading to a marked decrease in retinal inflammation [53]. This is evidenced by reduced levels of pro-inflammatory cytokines such as tumor necrosis factor-alpha (TNF-α) [54], interleukin-1 beta (IL-1β), and interleukin-6 (IL-6) [55], which are known to promote leukocyte adhesion and capillary occlusion. In parallel, MET mitigates oxidative stress by enhancing the activity of antioxidant enzymes including superoxide dismutase (SOD) [56] and catalase, and by reducing the generation of reactive oxygen species (ROS) within retinal endothelial cells and pericytes [57,58]. This antioxidant effect helps to prevent mitochondrial dysfunction, lipid peroxidation, and DNA damage, all of which are hallmarks of diabetic retinal injury. MET also exerts a protective influence on the integrity of the retinal vasculature by preserving tight junction proteins such as occludin [57] and claudin-5 [54], thereby reducing vascular permeability and preventing blood–retinal barrier disruption. Furthermore, MET suppresses the overexpression of vascular endothelial growth factor (VEGF), which plays a pivotal role in pathological neovascularization and macular edema in diabetic retinopathy (DR) [59]. This effect has been demonstrated in both early and advanced stages of DR in rodents, where MET reduced VEGF mRNA and protein levels in the retina and choroid [60]. Additionally, MET has demonstrated neuroprotective properties by reducing apoptosis in retinal ganglion cells and photoreceptors, possibly through the modulation of the Bcl-2/Bax ratio and inhibition of caspase-3 activation [55,61]. Morphological studies in diabetic rats have shown that MET prevents retinal thinning and preserves the organization of retinal layers, as assessed by histological analysis [54]. Altogether, these findings underscore the multifaceted role of MET in counteracting the complex pathophysiological mechanisms of diabetic retinal vasculature dysfunction. By targeting inflammation, oxidative stress, vascular leakage, and neuronal apoptosis, MET shows promise as a disease-modifying agent in the preclinical setting.

Preclinical studies have provided compelling evidence that MET may play a protective role in neovascular age-related macular degeneration (wet AMD) through its anti-angiogenic, anti-inflammatory, and antioxidant effects [62]. In laser-induced choroidal neovascularization models, which mimic wet AMD pathology, MET treatment has been shown to significantly reduce the size and leakage of neovascular lesions [63]. This effect is primarily mediated by activation of AMP-activated protein kinase (AMPK), which suppresses hypoxia-inducible factor-1α (HIF-1α) and downregulates vascular endothelial growth factor (VEGF), a central mediator of pathological angiogenesis in AMD [56,64]. Furthermore, MET directly impairs endothelial cell migration, proliferation, and tube formation in angiogenesis assays using human choroidal and umbilical vein endothelial cells exposed to VEGF or high-glucose conditions, further supporting its anti-angiogenic potential [65]. Collectively, these findings suggest that MET targets multiple pathogenic pathways relevant to neovascular AMD and may serve as a potential adjunct or alternative therapeutic strategy in managing this blinding disease, pending further translational and clinical investigation.

## 4. Novel Antihyperglycemic Agents in the Therapy of T2D

SGLT-2 inhibitors, such as empagliflozin (EMPA), dapagliflozin (DAPA), canagliflozin (CANA), and ertugliflozin (ERTU), are medications that block the sodium-glucose cotransporter 2 in the kidneys, thereby reducing glucose reabsorption and facilitating its excretion through urine [66]. These agents have demonstrated significant benefits in glycemic control, but their effects extend beyond this aspect. SGLT-2 inhibitors have shown a positive impact on renal and cardiovascular function, slowing the progression of kidney failure and reducing the risk of cardiovascular events [67,68]. Recent studies have also indicated hepatoprotective effects by reducing hepatic steatosis and improving the lipid profile [69]. ERTU helps control blood sugar in T2D while also providing cardiovascular and renal protection by lowering glucose levels, blood pressure, and reducing the risk of heart failure [70] and kidney damage [71].

GLP-1 receptor agonists are a class of medications that stimulate glucose-dependent insulin secretion and inhibit glucagon secretion [72]. In addition to their effects on glucose metabolism, these agents have been associated with weight loss and cardiovascular protection [73]. A significant additional benefit is the reduction in the risk of diabetic neuropathy and nephropathy, achieved through decreasing inflammation and oxidative stress [74,75]. Both semaglutide (SEMA) and liraglutide (LIRA) have also shown hepatoprotective effects, reducing hepatic steatosis and improving liver function [73].

TZP is an innovative agent that acts by stimulating both GLP-1 and GIP receptors, two polypeptides involved in regulating glucose and fat metabolism [76,77]. Studies have shown that TZP controls blood sugar, promotes weight loss, and has beneficial effects on heart and kidney function [78]. Additionally, this drug has been shown to protect the liver from hepatic steatosis and reduce the risk of progression to cirrhosis. Furthermore, there is evidence suggesting that TZP reduces oxidative stress and inflammation, two key processes in the pathogenesis of T2D [27,28,79].

## 5. Experimental Studies on Organ Protection Provided by Traditional and Novel Antidiabetic Medication

### 5.1. MET’s Effects

Various studies have provided valuable insights into the pathological mechanisms underlying DM. These investigations also explored the therapeutic potential of the traditional antidiabetic drug MET across a range of experimental settings. The compiled data from these studies are summarized in Table 1.

The effects of MET on the pancreas, liver, and kidneys were investigated in albino rats with alloxan-induced DM. Through histopathological examination of tissue samples stained with hematoxylin-eosin (H&E), they demonstrated that daily oral treatment with this antidiabetic drug alleviated structural lesions, supporting the hypothesis of its cytoprotective effect on major metabolic organs [80,81].

The impact of MET on the liver was also studied in rats with experimental DM induced by STZ. Histological results revealed a reduction in hepatic steatosis, while immunohistochemical analysis indicated a decrease in the expression of oxidative stress markers such as malondialdehyde (MDA), SOD, reduced glutathione (GSH), and catalase (CAT), suggesting an antioxidant effect of the treatment [82].

Other researchers examined the influence of this classic antidiabetic drug on the kidneys of rats with STZ-induced DM, observing a significant reduction in glomerular lesions through histopathological evaluations. Immunohistochemical analysis confirmed a decrease in the expression of inflammatory markers, such as TNF-α, IL-6, as well as transforming growth factor-beta (TGF-β1) and connective tissue growth factor (CTGF), indicating a potential protective effect on renal function [83,84].

The effects of MET on the myocardium were also studied in diabetic rats fed a high-fat diet and treated with STZ. Histopathological investigations revealed a reduction in myocardial fibrosis, while immunohistochemical analysis of H&E-stained sections showed a decrease in the expression of pro-fibrogenic markers such as inducible nitric oxide synthase (iNOS), mammalian target of rapamycin (mTOR), and tissue inhibitor of metalloproteinase-1 (TIMP-1), further confirming MET’s protective role against myocardial damage in DM [85].

Another study focused on the histopathological analysis of the myocardium, stained with H&E, in STZ-induced diabetic rats. This study demonstrated that MET administration resulted in a denser organization of myocardial fibers and a significant reduction in vacuolization compared to untreated diabetic animals, where myocardial fibers were arranged chaotically and exhibited vacuolization. Masson staining further highlighted a significant reduction in fibrotic changes after administration of the antidiabetic agent [86].

Niu et al. investigated retinal and aortic changes in genetically modified db/db mice (heterozygous for the Lepr gene mutation of leptin), analyzing the expression of specific apoptosis and autophagy markers (cadherin 5, platelet endothelial cell adhesion molecule—PECAM1/CD31, anti-LC3B antibodies, anti-GLI1, anti-ATG7) through electron microscopy, immunofluorescence, and Western blot analysis. They obtained clear evidence of endothelial and cardiac protective actions, demonstrated by improved autophagy activation at the vascular endothelial level and a pro-angiogenic effect [87].

The impact of MET on accelerated atherosclerosis in ApoE−/− and Myh11-cre-EGFP mice with STZ-induced DM and a high-fat diet was assessed through immunohistochemical analyses and Western blot, which revealed that MET modulated the expression of markers such as Pdlim5, AMPK, and SMA. This influenced the migration of vascular smooth muscle cells, reducing the progression of atherosclerotic lesions [88].

Another research explored the effects of MET on the articular capsule and visceral adipose tissue in db/db transgenic mice, showing a significant reduction in the expression of markers such as TGF-β1, Acta2, and Ccn2 and a decrease in posterior capsule thickness and collagen density. Additionally, MET administration increased the expression of adiponectin, indicating a potential anti-inflammatory, antioxidant, and antifibrotic effect in the context of diabetic musculoskeletal complications [89].

Srivastava and Goodwin utilized db/db and db/db;GRECKO transgenic mice (spontaneously developing DM) fed a high-fat diet to analyze the effect of MET on renal, cardiac, hepatic, and adipose tissues. Histopathological examination using Masson and Sirius Red staining revealed that MET reduced inflammation and fibrogenesis in the studied tissues, decreased the expression of immunohistochemical markers such as smooth muscle actin (α-SMA) and fibroblast-specific protein 1 (FSP-1), as well as inflammatory cytokines like IL-1β and IL-6, demonstrating the protective effects of this drug against organ damage in DM [16].

### 5.2. The Effects of SGLT-2 Inhibitors

SGLT-2 inhibitors (EMPA, DAPA, CANA, and ERTU) were examined for their diverse protective effects. These effects were primarily observed in organs commonly affected by T2D, such as the heart, kidneys, and liver, in various laboratory animal models (Table 2).

Han et al. demonstrated that EMPA improves revascularization in posterior limb ischemia in STZ-induced diabetic C57BL/6 mice fed a high-fat diet. This effect was linked to increased expression of GPX4 and PECAM-1, confirmed through immunofluorescence and polymerase chain reaction (PCR), suggesting a potential therapeutic role in treating ischemic diabetic complications [90].

Complex research has explored the effects of EMPA and CANA on the kidneys and pancreas in Akimba transgenic mice with DM. Histopathological examination using H&E, Periodic acid–Schiff (PAS), and Masson staining, along with immunohistochemical analysis utilizing anti-SGLT-2 antibodies, revealed that these SGLT-2 inhibitors positively affect the pancreas, increasing islet mass and plasma insulin levels. They also benefit the kidneys by ameliorating structural lesions and reducing hypertrophy in diabetic animals [91].

Two studies investigated the effects of DAPA on the kidneys of Wistar rats [92] and C57BL/6 transgenic mice [93] with STZ-induced T2D, showing that this SGLT-2 inhibitor prevents functional and structural renal degradation. It was found to reduce albuminuria, with histopathological analysis (PAS staining) revealing attenuated tubulo-interstitial lesions in diabetic rats [92]. In diabetic mice, it reduced renal inflammation, fibrosis, glomerular lesions, and oxidative stress (PAS and Masson staining) [93]. Additionally, ERTU demonstrated improvements in renal structural lesions, evidenced histopathologically and immunohistochemically, by maintaining podocyte integrity, reducing mesangial expansion, glomerular basement membrane thickness, and glomerular volume in C57BL/6J mice with high-fat diet-induced DM [94].

Other studies focusing on the pancreas confirmed that EMPA exerts protective effects at the pancreatic level. Histological and immunohistochemical analyses revealed a reduction in pathological changes and inflammatory cell infiltration, as well as a decrease in the expression of inflammatory markers related to pyroptosis, including NLPR3 (NOD-like receptor pyrin domain-containing), caspase-1, and gasdermin D (GSDMD) [95].

Another research group assessed the effects of ERTU on the heart in an experimental DM model in transgenic C57BL/6J mice fed a high-fat diet and excess sucrose. For myocardial cross-sectional area evaluation, H&E staining was used, while Picrosirius Red staining assessed fibrosis. Immunohistochemical analysis utilized the lipid peroxidation marker 4-hydroxynonenal. Histopathological examination revealed that ERTU exhibited cardioprotective effects by preventing myocardial structural alterations and cardiac remodeling, as well as reducing the expression of oxidative stress markers [96].

### 5.3. The Effects of GLP-1 Agonists

In recent years, the effects of two GLP-1 receptor agonists (SEMA and LIRA) have been extensively evaluated. These studies utilized transgenic mice and diabetic rodent models to assess their potential benefits. The experimental outcomes are detailed in Table 3.

The effects of SEMA on the kidneys, liver, and epididymal adipose tissue in db/db mice, C57BL/6J mice, and Zucker diabetic fatty (ZDF) obese rats were studied using histological analyses with H&E, PAS, Sirius red, and Masson stains, as well as RNA sequencing. The results revealed a significant reduction in inflammation and fibrosis, accompanied by an increase in the expression of genes related to fibrotic and inflammatory factors, while a marked decrease in genes associated with mitochondrial function was observed. These findings suggest favorable effects of this GLP-1 agonist in diabetic nephropathy [97].

Iwai et al. analyzed mitochondrial signaling and GLP-1 receptor expression in the liver and gastrocnemius muscle of KK-Ay diabetic mice on a diet supplemented with dietoxicarbonyl-1,4-dihydrocolidine, using biochemical tests, mitochondrial oxygen consumption measurements, and histological examination. SEMA was shown to increase the production of insulin-like growth factor 1 (IGF-1), reduce pro-inflammatory cytokines, and decrease reactive oxygen species (ROS) accumulation. Furthermore, SEMA improved skeletal muscle atrophy by directly stimulating GLP-1R in myocytes, confirming its protective action on skeletal muscle damage in chronic liver injury in diabetic animals [98].

Another study investigated the effects of SEMA in BKS db/db mice, which are leptin receptor-deficient, hyperphagic, and diabetic. The use of this antihyperglycemic agent significantly reduced blood glucose, body weight, and serum markers of liver dysfunction. Histological analysis revealed that SEM treatment markedly improved liver architecture, reducing steatosis, hepatocellular ballooning, and intrahepatic triglycerides. Additionally, treatment improved the hepatic expression of markers of de novo lipogenesis, indicating a reduction in the synthesis of new fatty acids in the liver [99].

Li assessed the effects of SEMA on hepatic steatosis and dyslipidemia in C57BL/6J mice with STZ-induced DM and a high-fat diet, using histological techniques and glucose tolerance tests. Significant improvements in liver function were reported, highlighting the importance of this treatment in preventing hepatic complications in DM [100].

Other researchers examined the impact of chronic LIRA administration on pancreatic islets in ZDF rats, analyzing histological and functional changes. Treatment was found to improve β-cell function, increasing insulin secretion and content in the islets. However, LIRA did not maintain the normal architecture of pancreatic islets. A reduction in the number of α-cells and the area occupied by them was observed, along with selective mitochondrial damage in α-cells. These changes suggest that while LIRA protects β-cell function, it may disrupt the hormonal balance of pancreatic islets [101].

The protective effects of LIRA on pancreatic and hepatic structural alterations have also been demonstrated histologically and immunohistochemically in a spontaneous DM model in KK/Upj-Ay/J (KKAy) transgenic mice. These effects were evidenced by the normalization of mitochondrial morphology, rough endoplasmic reticulum, Golgi apparatus, and an increase in the number of secretory granules in the beta cells of the Langerhans islets, as well as by the increased expression of glucose transporter 4 (GLUT4) in the liver [102].

Literature data highlight the cardioprotective and antioxidant actions of LIRA in Wistar rats with STZ-induced DM and a high-fat diet. LIRA treatment was associated with increased levels of cardiac biomarkers, including troponin I and creatine kinase-MB, and enhanced antioxidant enzyme activities, such as GPx and SOD. Histological studies, Western blot, and immunohistochemical analyses showed that LIRA mitigated cytoplasmic and nuclear degeneration in myocardial cells, increased levels of markers like ILK, P-PI3K, P-AKT, and BCL2, and reduced levels of caspase 3, BAX, and P-PTEN, indicating a reduction in cardiomyocytes apoptosis [103].

A comprehensive study investigated the effects of combining ramipril (an angiotensin-converting enzyme inhibitor), EMPA, and SEMA on the progression of diabetic kidney disease and cardiac impairment in db/db mice with T2D, following uninephrectomy. SEMA treatment decreased hyperglycemia, albuminuria, glomerular hyperfiltration, and mesangial matrix expansion. In the kidneys, the triple treatment reduced the expression of pro-inflammatory genes, such as Ccl2, and profibrotic genes, including TGF-β1. Histopathological examination with PAS and Picrosirius Red stains showed that the combination of EMPA and SEMA, alongside the inhibition of the renin–angiotensin–aldosterone system, significantly reduced cardiomyocytes hypertrophy and cardiac fibrosis, confirming the effectiveness of combining these medications for cardiac and renal protection [104].

### 5.4. TZP’s Effects

During 2024 and 2025, a number of preclinical studies were conducted using mice and rat models to investigate the effects of TZP. These studies focused on its ability to improve the function of key metabolic organs, including the liver, pancreas, and kidneys (Table 4).

Tian et al. have reported findings regarding the effects of SEM and TZP on diabetic nephropathy in C57BL/6J mice with STZ-induced T2D and a high-fat diet. Indicators of diabetic kidney injury were evaluated, including the urinary albumin-to-creatinine ratio, markers of oxidative stress (GPx, SOD, and CAT), and histological analysis with H&E and Masson stains, as well as immunohistochemical evaluation using antibodies against p-PI3K and p-AKT. The research demonstrated that both TZP and SEM exhibit antioxidant effects and improve renal dysfunction and structural damage in diabetic animals [105].

A comparative study investigated the effects of TZP and SEMA on the pancreas and liver in a T2D and obesity model in db/db mice, with TZP showing stronger organ-protective effects than the GLP-1 analog. Histologically, TZP treatment increased pancreatic β-cell mass and improved insulin granules, suggesting protection and regeneration of insulin-secreting cells. In the liver, TZP more effectively than SEMA reduced lipid accumulation, evidenced by Oil Red O staining, decreased hepatic inflammation by improving the M1/M2 macrophage ratio, and lowered the liver-to-spleen ratio, indicating an improvement in hepatic steatosis. These findings suggest that both antihyperglycemic agents, particularly TZP, not only enhance insulin secretion but also protect the liver from fat accumulation and inflammation [106].

Yuan et al. evaluated the effects of TZP in db/db and C57BL/6J diabetic mice following STZ administration and a high-fat diet, using histological techniques, including H&E and Sirius Red staining, qPCR, and Western blot analysis. TZP treatment significantly reduced hepatocyte steatosis and inflammatory infiltration and increased the expression of GLP-1 and GIP receptors, without significantly affecting liver tissue fibrosis [107].

The protective effects of TZP on metabolic steatohepatitis, liver fibrosis, and hepatocarcinogenesis were demonstrated in C57BL/6J mice with STZ-induced DM and a high-fat diet. Histologically, TZP treatment reduced hepatic steatosis, inflammation, and fibrosis in the early and intermediate stages of the disease. It also significantly decreased the occurrence of liver tumors, with no tumors detected in some treated cases. Transcriptomic analysis revealed that TZP regulated the expression of genes involved in fatty acid degradation, gluconeogenesis, and the cell cycle, while inhibiting genes associated with fibrogenesis and Wnt signaling. These results suggest that TZP may slow the progression of metabolic steatohepatitis, reduce fibrosis, and prevent hepatocarcinogenesis [108].

In a recent study, Yang et al. highlighted the protective, antioxidant, and anti-inflammatory effects of TZP on diabetic nephropathy in an STZ-induced DM model in rats. Histological examination of kidney tissue using H&E and PAS staining showed that TZP treatment reduced tubular and glomerular lesions, as well as renal apoptosis levels [109].

## 6. Limitations

While the preclinical studies reviewed in this manuscript provide valuable insights into the potential protective effects of antidiabetic agents, several limitations inherent in these studies must be considered when interpreting the results. One of the primary challenges in translating findings from animal models to human clinical applications is the heterogeneity of preclinical models. Animal models of DM are highly variable in terms of strain, age, sex, and the method of induction of DM (e.g., STZ, high-fat diet, or genetic models). These variations introduce significant biological variability in the response to treatment, making it difficult to generalize results across different studies. For example, some rodent strains may exhibit greater susceptibility to vascular dysfunction than others, which can skew the observed efficacy of antidiabetic agents. Additionally, age and sex differences can influence both the progression of diabetic complications and the drug response, but these factors are often not adequately controlled or reported in many studies.

Moreover, a non-standardized methodology in histological and functional assessments complicates the interpretation of results. Variations in the preparation of tissue samples, staining techniques, and imaging protocols can introduce observer bias and lead to inconsistent reporting of outcomes, such as retinal thickness, microvascular integrity, and inflammatory markers. These methodological inconsistencies are particularly evident in studies examining the retinal vasculature, where the assessment of vascular leakage, neovascularization, and blood–retinal barrier disruption can vary significantly based on the imaging modality used (e.g., fluorescein angiography, optical coherence tomography, or confocal microscopy). Without standardized protocols, it is difficult to draw definitive conclusions about the relative effectiveness of different antidiabetic agents in mitigating diabetic retinopathy or other diabetic complications.

Despite these limitations, the body of research presented highlights the promising potential of antidiabetic agents, particularly MET and newer therapies, in protecting against diabetic complications. However, it is essential for future studies to employ more rigorous, standardized methodologies, control for confounding variables such as age and sex, and investigate the long-term safety and efficacy of these treatments. Addressing these issues will be crucial in translating the positive results seen in animal models into clinically relevant therapies for patients with DM.

## 7. Future Perspectives and Clinical Implications

Future research on the histological effects of traditional antidiabetic medications and new antihyperglycemic agents presents significant opportunities to deepen our understanding of their impact on tissue structure and function in T2D and its complications. A key objective would be the development of more representative preclinical models that better reflect the complexity of diabetic pathologies and associated comorbidities, such as non-alcoholic steatohepatitis, diabetic nephropathy, and diabetic cardiomyopathy. Current animal models are often limited in their ability to fully capture the diversity and complexity of treatment responses observed in human patients. Advances in this area could help generate more clinically relevant data. For example, the use of advanced imaging techniques, such as electron microscopy or real-time tissue imaging, could enable precise evaluation of subtle histological changes at the cellular and molecular levels, which might otherwise go unnoticed in conventional studies.

Another important focus for future research would be the standardization of histological evaluation methodologies and relevant parameters for different diabetic complications. Currently, there is significant variability in how research groups measure and interpret histological changes, making it difficult to directly compare results between studies. Establishing uniform protocols for evaluating tissue lesions such as hepatic steatosis, fibrosis, inflammation, and apoptosis would help improve consistency and the validity of studies. Additionally, conducting extensive studies would be essential to assess the effects of chronic treatments on target organ health and to determine whether positive histological changes observed in the early stages of treatment are sustained long-term.

The clinical implications of this research could be profound. First, by enhancing our understanding of the mechanisms through which medications affect tissue structure at the microscopic level, it may be possible to identify the most effective therapeutic options for preventing and treating complications of T2D. For instance, TZP could offer a promising solution for improving hepatic and renal histology, significantly impacting hepatic fat accumulation, fibrosis, and hepatic tumorigenesis, as demonstrated in animal studies. Similarly, the use of antihyperglycemic agents that can reduce oxidative stress and inflammation may help protect target organs, such as the kidneys and liver, from damage caused by DM. Furthermore, these studies could aid in the development of personalized therapeutic strategies, where treatments are chosen not only based on their effectiveness in controlling blood glucose but also according to the histological response of individual patients, potentially improving disease management.

As the treatment of diabetic complications moves toward more individualized care, it is essential to recognize the role that preclinical models play in advancing this personalized medicine paradigm. The variability inherent in these models, such as differences in genetic background, age, sex, and dietary factors, offers an opportunity to explore how these factors influence the efficacy of antidiabetic agents. By utilizing a diverse range of rodent models, researchers can better simulate the heterogeneous patient populations seen in clinical practice, allowing for the identification of biomarkers and the optimization of treatment strategies that cater to individual patient needs. Preclinical studies that account for these variations can provide insights into how specific subgroups of patients, based on their genetic makeup, underlying health conditions, or comorbidities, might respond to different therapeutic agents. Furthermore, these models can help predict potential adverse effects or treatment resistance in certain patient subsets, guiding the development of safer and more effective therapies.

The integration of findings from preclinical models into clinical research can facilitate a more tailored approach, ensuring that therapies are not only effective but also appropriate for the individual patient’s specific profile. As such, preclinical models play a pivotal role in advancing the personalized approach to DM treatment, ultimately leading to better-targeted therapies that enhance efficacy while minimizing risks.

Moreover, advances in the research of histological effects of antidiabetic medications could contribute to reducing the risk of progression to more severe stages of DM, such as renal failure, liver cirrhosis, or heart failure. By reducing inflammation and oxidative stress in affected tissues, treatments could prevent irreversible organ damage and reduce the risk of major complications associated with T2D.

In the future, integrating these findings into clinical practice could lead to a more targeted approach in T2D treatment, where antihyperglycemic agents are prescribed not only based on their effectiveness in controlling blood glucose but also on their ability to prevent or ameliorate tissue damage associated with the disease.

## 8. Conclusions

T2D has a significant negative impact on various organs, particularly the pancreas, kidneys, liver, cardiovascular system, and nervous system. Chronic hyperglycemia, oxidative stress, and inflammation play a central role in the histological damage of these organs.

As a traditional antidiabetic agent, MET has shown protective effects on the pancreas, liver, kidneys, and heart. Histopathological and immunohistochemical studies have confirmed its ability to reduce oxidative stress, inflammation, and fibrosis in various tissues. SGLT-2 inhibitors and GLP-1 receptor agonists have also demonstrated significant protective effects on organs affected by T2D. These agents not only improve glycemic control but also slow the progression of diabetic complications through anti-inflammatory, antioxidant, and antifibrotic mechanisms. Compared to other antihyperglycemic agents, TZP has shown superior effects in protecting the pancreas, liver, and kidneys. Histological studies have indicated beta-cell regeneration in the pancreas, a reduction in hepatic steatosis, and renal protection against diabetic-induced damage.

Although animal studies show promising results, caution is required when extrapolating these findings to humans. Variations in experimental models, dosages, and treatment durations may influence the conclusions. Future research should focus on large-scale clinical trials to validate these benefits and optimize therapeutic strategies.

Identifying drugs with organ-protective effects could transform the management of T2D, shifting the focus from merely controlling blood glucose to preventing and treating diabetic complications at the histological level. Personalizing treatment based on tissue responses in patients could significantly improve their prognosis and quality of life.

## Figures and Tables

**Table 1:** MET’s effects in animal models of T2D.

Article	Animals	Study Design	Pathology Model	Biochemical, Histological, Immunohistochemical Tests	Organs	Drug (Dose, Duration of Drug Exposure)	Other Drugs
Almuttairi, 2023 [80]	Albino rats	In vivo, randomized	Alloxan-induced T2D	Markers: None reported. Histology: H&E	Pancreas Liver Kidney	MET (1000 mg/kg daily, oral gavage, 4 weeks)	-
Salem, 2022 [81]	Albino rats	In vivo, randomized	Alloxan-induced T2D	Markers: Glucose, AST, ALT, ALP, Urea, Creatinine, Uric acid. Histology: H&E	Pancreas Liver Kidney	MET (45 mg/kg/day, oral, 28 days). MET nanoparticles (45 mg/kg/day, oral, 28 days)	-
Mobasher et al., 2020 [82]	Sprague Dawley albino rats	In vivo, randomized	STZ-induced DM	Markers: SOD, CAT, GSH, MDA, ALT, AST, ALP, arginase, albumin, total bilirubin, TP, PCNA, caspase-3. Histology: H&E, ICH (PCNA, caspase-3).	Liver	MET (150 mg/kg, oral gavage, every other day for 100 days)	-
Dallak et al., 2018 [83]	Albino rats	In vivo, randomized	HFD + STZ-induced T2DN	Markers: Glucose, urea, creatinine, TNF-α, hs-CRP. Histology: H&E, PAS.	Kidney	MET (200 mg/kg/day, oral gavage, started 2 weeks before STZ and continued for 12 weeks)	-
Zhang et al., 2017 [84]	Wistar rats	In vivo, randomized	HFD + low-dose STZ-induced T2DN	Markers: TGF-β1, CTGF, SOD, MDA, GSH-Px, FBG, HDL-c, LDL-c, TG, TC, BUN, SCr, UAER. Histology: H&E, ICH, TEM.	Kidney	MET (70 mg/kg/day, oral gavage, 13 weeks)	Captopril (10 mg/kg/day, oral gavage, 13 weeks)
Dawood et al., 2022 [85]	Wistar rats	In vivo, randomized	HCFD + STZ-induced T2D-induced DCM	Markers: Desmin, iNOS, AMPK, mTOR, TIMP-1, collagen. Histology: H&E, Masson’s trichrome, TEM, ICH, Western blot, qPCR, ECG.	Heart (left ventricle)	MET (200 mg/kg/day, oral, started day 1, continued for 12 weeks)	-
Yang et al., 2020 [86]	Male C57BL/6J mice	In vivo and in vitro, randomized	STZ-induced DCM	Markers: LDH, AST, CK, TC, TG, Bax, Bcl-2, PK2, PKR1, PKR2, p-AKT, AKT, p-GSK3β, GSK3β. Histology: H&E, Masson’s trichrome, TUNEL, ICH, electron microscopy, RT-qPCR, Western blot.	Heart	MET (250 mg/kg/day, oral in drinking water, 16 weeks).	-
Niu et al., 2019 [87]	db/db mice	In vivo, randomized	TMM	Markers: PECAM1/CD31, GLI1, PTCH1, SMO, HHIP, LC3-II, CASP3, CASP8, CASP9, BECN1, BNIP3, BCL2. Histology: Immunofluorescence, electron microscopy, Western blot, TUNEL assay, luciferase assay.	Retina Aorta	MET (300 mg/kg daily, i.p. injection, 4 weeks)	-
Yan et al., 2021 [88]	ApoE−/− mice, Myh11-cre-EGFP mice	In vivo, randomize	STZ + HFD-induced diabetes-accelerated atherosclerosis model	Markers: Pdlim5, AMPK, α-SMA, Oil Red O staining, phosphorylated Pdlim5. Histology: ICH, Western blotting, en face aortic lesion analysis.	Aorta—en face lesion analysis, Oil Red O staining. Carotid artery—wire injury model to study vascular smooth muscle cell (VSMC) migration.	MET (300 mg/kg daily, oral gavage, 8 weeks)	-
Naito et al., 2024 [89]	db/db mice, WT mice	In vivo, randomized	TMM	Markers: Col1a1, Col1a2, Col3a1, Acta2, Ccn2, TGF-β1. Histology: H&E, PSR, ICH for TGF-β1, Acta2, Ccn2.	Knee joint capsule Visceral fat (for adiponectin expression analysis)	MET (100 mg/kg daily, i.p. injection, 4, 8 weeks)	-
Srivastava and Goodwin, 2023 [16]	db/db mice, db/db; GRECKO mice	In vivo, randomized	TMM	Markers: IL-6, Wnt3a, α-SMA, FSP-1, Axin2. Histology: Masson’s trichrome stain, PSR, ICH, qPCR, cytokine array (IL-1β, IL-6, IFN-γ).	Kidney Heart Liver Adipose tissue	MET (100 mg/kg daily, oral gavage, for 8 weeks)	Wnt inhibitor (LGK974, 5 mg/kg, oral gavage, 6 doses per week for 8 weeks).

DCM: Diabetic Cardiomyopathy; DM: Diabetes Mellitus; H&E: Hematoxylin and Eosin Staining; HCFD: High-Carbohydrate, High-Fat Diet; HFD: High-Fat Diet; ICH: Immunohistochemistry; MET: Metformin; PSR: Picrosirius Red Staining; STZ: Streptozotocin; T2D: Type 2 Diabetes, T2DN: Type 2 Diabetic Nephropathy; TEM: transmission electron microscopy; TMM: Transgenic Mice Model; WT: Wild-Type.

**Table 2:** SGLT-2 inhibitors effects in animal models of T2D.

Article	Animals	Study Design	Pathology Model	Biochemical, Histological, Immunohistochemical Tests	Organs	Drug (Dose, Duration of Drug Exposure)	Other Drugs
Han et al., 2023 [90]	C57BL/6 mice	In vivo, randomized	STZ + HFD-induced HLI	Markers: GPX4, PECAM-1, α-SMA, 4-HNE. Histology: Immunofluorescence, Western blot, qRT-PCR, TEM.	Skeletal muscle (gastrocnemius) Hindlimb vasculature	EMPA (10 mg/kg, intramuscular injection, every 3 days for 21 days)	-
Matthews et al., 2022 [91]	Kimba and Akimba mice	In vivo, randomized	TMM	Markers: SGLT2, insulin. Histology: H&E, PAS, Masson’s trichrome staining, ICH, Western blot, kidney-to-body weight ratio.	Kidney Pancreas	DAPA, EMPA, CANA (25 mg/kg/day, oral, in drinking water, 8 weeks)	-
Farias et al., 2023 [92]	Wistar rats	In vivo, randomized	STZ-induced T2D, early-stage DKD	Markers: Albumin, β2-microglobulin, LDH, γ-GT, megalin. Histology: PAS staining, immunofluorescence, SDS-PAGE, Western blot, renal histomorphometry.	Kidney	DAPA (1 mg/kg/day, oral gavage, started 1 day post-STZ, continued for 8 weeks)	-
Dia et al., 2023 [93]	C57BL/6 and FVB/NJ mice	In vivo, randomized	HFD + STZ-induced T2D, DKD	Markers: CYP4A, 20-HETE, MCP-1, IL-1β, IL-6, IL-17, TNFα, fibronectin, collagen IV. Histology: PAS, Masson’s trichrome, Western blot, RT-PCR, ELISA, HPLC, NADPH oxidase assay.	Kidney	DAPA (1.5 mg/kg/day, i.p., 8 weeks). HET0016 (5 mg/kg/day, s.c., 10 weeks)	Insulin (2 IU/day, i.p., 8 weeks).
Kim et al., 2022 [94]	C57BL/6J mice	In vivo, randomized	HFD-induced early DKD	Markers: Albuminuria, SGLT2 (mRNA and protein), glomerular volume, mesangial expansion, GBM thickness. Histology: ICH, renal histomorphometry.	Kidney (renal cortex)	ERTU (mixed with HFD, oral, 16 weeks)	-
Liu et al., 2021 [95]	db/db mice	In vivo and in vitro, randomized	TMM	Markers: NLRP3, caspase-1, GSDMD. Histology: H&E, ICH, immunofluorescence, Western blot (pancreas and β TC-6 cells).	Pancreas	EMPA (10 mg/kg/day, oral gavage, 6 months)	-
Croteau et al., 2021 [96]	C57BL/6J mice	In vivo, randomized	HFHS induced DCM	Markers: HbA1c, glucose, insulin, HOMA-IR, ROS (4-HNE), ATP, phosphocreatine/ATP. Histology: H&E, PRS, ICH, 4-HNE staining, 31P-NMR spectroscopy, RNA-seq.	Heart (left ventricle)	ERTU (0.5 mg/g of diet, oral, 4 months)	-

CANA: Canagliflozin; DAPA: Dapagliflozin; DCM: Diabetic Cardiomyopathy; DKD: Diabetic Kidney Disease; EMPA: Empagliflozin; ERTU: Ertugliflozin; H&E: Hematoxylin and Eosin Staining; HFD: High-Fat Diet; HFHS: High-Fat, High-Sucrose; HLI: Diabetic Hindlimb Ischemia; ICH: Immunohistochemistry; PRS: Picrosirius Red; T2D: Type 2 Diabetes; TEM: transmission electron microscopy; TMM: Transgenic Mice Model.

**Table 3:** GLP-1 agonists effects in animal models of T2D.

Article	Animals	Study Design	Pathology Model	Biochemical, Histological, Immunohistochemical Tests	Organs	Drug (Dose, Duration of Drug Exposure)	Other Drugs
Zhao et al., 2024 [97]	db/db mice, UUO mice, ZDF rats	In vivo, randomized	TMM	Markers: GLP-1R, GCGR, TGF-β1, α-SMA, COL1A1, NF-κB, IL-1β, TNF-α, PGC-1α, PGC-1β. Histology: H&E, Masson’s trichrome, Sirius Red, ICH, Western blot, qPCR, RNA sequencing, mitochondrial OCR assay.	Kidney, Liver, Epididymis adipose tissue	SEMA (120 mg/kg/day, s.c. for 8 weeks)	-
Iwai et al., 2023 [98]	Diabetic KK-Ay mice	In vivo, randomized	TMM	Markers: GLP-1R, PGC-1α, PKA, AKT, NF-κB, MSTN, MyoG, MyoD, MuRF-1. Histology: H&E, Sirius Red, Immunofluorescence, Western blot, qRT-PCR, mitochondrial OCR assay.	Liver Gastrocnemius muscle	SEMA (3 nmol/kg, s.c., every 3 days for 6 weeks)	-
Soto-Catalán et al., 2024 [99]	BKS db/db mice	In vivo, randomized	TMM	Markers: GLP-1R, ALT, AST, AP, triglycerides, total cholesterol, c-HDL, SCD1, Acaca, Fasn. Histology: H&E, Oil Red O staining, DXA body composition, qPCR, Western blot, lipidomic analysis.	Liver	SEMA (25 µg/kg/week for 2 weeks, then 100 µg/kg/week for 9 weeks, s.c.)	-
Li et al., 2022 [100]	C57BL/6J mice	In vivo, randomized	STZ + HFD-induced T2D and NAFLD	Markers: ABHD6, miR-5120, GLP-1R, CD36, PPARγ, PPARα, ALT, AST, TG, TC, FFA, LDL-C, HDL-C. Histology: H&E, Sirius Red, Oil Red O staining, qPCR, Western blot, ELISA, OGTT, IPITT, dual-luciferase assay.	Liver	SEMA (0.42 mg/kg/week, s.c., for 12 weeks)	-
Schwasinger-Schmidt et al., 2013 [101]	ZDF rats	In vivo and in vitro, randomized	TMM	Markers: Insulin, Proinsulin, Glucagon, Somatostatin, Ki-67, CD34. Histology: H&E, ICH, immunofluorescence, electron microscopy, insulin ELISA, islet density, morphology, granule analysis.	Pancreas	LIRA (0.225 µg/g, s.c., twice daily for 9 weeks)	-
Chen et al., 2013 [102]	Male KKAy mice and C57BL/6J (C57) mice	In vivo, randomized	TMM	Markers: GLUT4, keletal muscle pyruvate kinase, hexokinase, TEM, RNA isolation, real-time PCR.	Liver, Skeletal muscle	LIRA (250 μg/kg/day, s.c., 6 weeks)	-
Alobaid et al., 2024 [103]	Wistar albino rats	In vivo, randomized	STZ + HFD-induced T2D and DCM	Markers: Troponin I, CK-MB, ILK, PI3K, AKT, PTEN, BCL2, BAX, caspase 3, MDA, SOD, GPx. Histology: H&E, TUNEL assay, ICH, ELISA, Western blot.	Heart	LIRA (150 µg/kg, s.c., twice daily for 3 weeks after 3 weeks of vehicle)	-
Martos-Guillami et al., 2024 [104]	db/db mice	In vivo, randomized	TMM	Markers: Ccl2, TGF-β1, ACE2, ACE, FABP4, IGFBP4, Myh7, WT1. Histology: H&E, PAS, PRS, IHC, qPCR, Western blot, GFR, albuminuria, CT.	Kidney Heart	SEMA (10 nmol/kg, s.c., twice weekly, 8 weeks) EMPA (10 mg/kg/day, oral gavage, 5 days/week, 8 weeks)	Ramipril (8 mg/kg/day, drinking water, 8 weeks)

CT: Computed Tomography; DCM: Diabetic Cardiomyopathy; EMPA: Empagliflozin; GFR: Glomerular Filtration Rate; H&E: Hematoxylin and Eosin Staining; HFD: High-Fat Diet, IHC: Immunohistochemistry; LIRA: Liraglutide; NAFLD: Non-Alcoholic Fatty Liver Disease; OCR: Oxygen Consumption Rate; PRS: Picrosirius Red; SEMA: Semaglutide; STZ: Streptozotocin; T2D: Type 2 Diabetes; TEM: Transmission Electron Microscopy; TMM: Transgenic Mice Model; ZDF: Zucker Diabetic Fatty Rats.

**Table 4:** TZP’s effects in animal models of T2D.

Article	Animals	Study Design	Pathology Model	Biochemical, Histological, Immunohistochemical Tests	Organs	Drug (Dose, Duration of Drug Exposure)	Other Drugs
Tian et al., 2025 [105]	C57BL/6J mice	In vivo, randomized	STZ + HFD-induced T2DN	Markers: PI3K, AKT, p-PI3K, p-AKT, GPX4, SOD, CAT. Histology: H&E, Masson’s trichrome, ICH, Western blot, RNA sequencing, oxidative stress enzyme assays.	Kidney	TZP (10 nmol/kg, i.p., daily for 2 weeks) SEMA (30 nmol/kg, i.p., daily for 2 weeks)	Insulin glargine (0.5 IU/day, s.c., for 2 weeks)
Iwamoto et al., 2024 [106]	db/db mice	In vivo, randomized	TMM	Markers: Blood glucose, insulin, glucagon, ALT, AST, LDH, insulin-related genes (Ins1, Ins2, Pdx-1, MafA, NeuroD, Munc18), Chemokine and cytokine genes (Ccl3, Ccl4, Ccl5, Tnf-α, IL-1β, Mcp1). Histology: H&E, Oil Red O, Azan, immunofluorescence, TEM, qPCR, flow cytometry, CT imaging.	Pancreas Liver	TZP (30 nmol/kg, s.c., twice weekly, 4 weeks) SEMA (200 nmol/kg, s.c., twice weekly, 4 weeks)	-
Yuan et al., 2024 [107]	db/db mice, STZ + HFD-induced C57BL/6 mice	In vivo, randomized	TMM STZ + HFD-induced	Markers: GLP-1R, GIPR, ALT, AST, TC, TG, LDL, HDL. Histology: H&E, Sirius Red staining, qPCR, Western blot, ELISA, GTT, lipidomic analysis.	Liver	TZP (0.15 mg/kg, s.c., every 3 days, for 31 days)	BGM0504 (0.05, 0.15, 0.5 mg/kg, s.c., every 3 days, for 31 days)
Jeong et al., 2024 [108]	C57BL/6J mice	In vivo, randomized	STZ + HFD-induced MASLD (including MASH, fibrosis, HCC)	Markers: ALT, AST, NAS score, fibrosis stage, triglycerides, gene expression via RNA-seq. Histology: H&E, Masson’s trichrome, in vivo liver imaging, ICH, transcriptomics, ATAC-seq.	Liver	TZP (dose not specified, administered for 10–11 weeks at stages 21–32 weeks, 28–38 weeks, 41–52 weeks)	-
Yang et al., 2025 [109]	BALB/c mice	In vivo, randomized	STZ-induced T2DN	Markers: BUN, sCr, AGEs, insulin, SOD, CAT, MDA, IL-1β, IL-6, TNF-α, NGAL, podocin, cystatin C, IL-17A, IL-17F, GLP-1R, Bax, Bcl-2. Histology: H&E, PAS, TUNEL, RT-qPCR, Western blot, ELISA, renal histomorphometry.	Kidney	TZP (3 and 10 nmol/kg/day, i.p., 8 weeks)	-

GTT: Glucose Tolerance Test; H&E: Hematoxylin and Eosin Staining; HCC: Hepatocellular Carcinoma; HFD: High-Fat Diet; IHC: Immunohistochemistry; MASH: Metabolic Dysfunction-Associated Steatohepatitis; MASLD: Metabolic Dysfunction-Associated Steatotic Liver Disease; SEMA: Semaglutide; STZ: Streptozotocin; T2D: Type 2 Diabetes; T2DN: Type 2 Diabetic Nephropathy; TEM: Transmission Electron Microscopy; TMM: Transgenic Mice Model; TZP: Tirzepatide.

## Data Availability

No new data were created or analyzed in this study. Data sharing is not applicable to this article.

## References

[1] Guo H., Wu H., Li Z. (2023). The pathogenesis of diabetes. Int. J. Mol. Sci..

[2] Durruty P., Sanzana M., Sanhueza L. (2019). Pathogenesis of Type 2 Diabetes Mellitus.

[3] Skyler J.S., Bakris G.L., Bonifacio E., Darsow T., Eckel R.H., Groop L., Groop P.-H., Handelsman Y., Insel R.A., Mathieu C. (2017). Differentiation of diabetes by pathophysiology, natural history, and prognosis. Diabetes.

[4] Hayden M.R. (2023). Overview and new insights into the metabolic syndrome: Risk factors and emerging variables in the development of type 2 diabetes and cerebrocardiovascular disease. Medicina.

[5] Zhao X., An X., Yang C., Sun W., Ji H., Lian F. (2023). Crucial role and mechanism of insulin resistance in metabolic disease. Front. Endocrinol..

[6] Baro B., Pegu R., Hazarika B., Sarma U., Rabha G., Kalita S. (2024). A study of histopathological changes in diabetic and nondiabetic cadaveric pancreas with respect to diabetic status. J. Cardiovasc. Dis. Res..

[7] Burns C., Francis N., Ahima R.S. (2023). Type 2 Diabetes-etiology, epidemiology, pathogenesis, treatment. Metabolic Syndrome.

[8] Rojas A., Lindner C., Schneider I., Gonzalez I., Uribarri J. (2024). The RAGE axis: A relevant inflammatory hub in human diseases. Biomolecules.

[9] Pedreanez A., Robalino J., Tene D., Salazar P. (2024). Advanced glycation end products of dietary origin and their association with inflammation in diabetes—A minireview. Endocr. Regul..

[10] Yang T., Qi F., Guo F., Shao M., Song Y., Ren G., Linlin Z., Qin G., Zhao Y. (2024). An update on chronic complications of diabetes mellitus: From molecular mechanisms to therapeutic strategies with a focus on metabolic memory. Mol. Med..

[11] Liu H., Wang X., Gao H., Yang C., Xie C. (2023). Physiological and pathological characteristics of vascular endothelial injury in diabetes and the regulatory mechanism of autophagy. Front. Endocrinol..

[12] Sanches J.M., Na Zhao L., Salehi A., Wollheim C.B., Kaldis P. (2023). Pathophysiology of type 2 diabetes and the impact of altered metabolic interorgan crosstalk. FEBS J..

[13] Bodhini D., Morton R.W., Santhakumar V., Nakabuye M., Pomares-Millan H., Clemmensen C., Fitzpatrick S.L., Guasch-Ferre M., Pankow J.S., Ried-Larsen M. (2023). Impact of individual and environmental factors on dietary or lifestyle interventions to prevent type 2 diabetes development: A systematic review. Commun. Med..

[14] Gancheva S., Jelenik T., Álvarez-Hernández E., Roden M. (2018). Interorgan metabolic crosstalk in human insulin resistance. Physiol. Rev..

[15] Casado M.E., Collado-Pérez R., Frago L.M., Barrios V. (2023). Recent advances in the knowledge of the mechanisms of leptin physiology and actions in neurological and metabolic pathologies. Int. J. Mol. Sci..

[16] Srivastava S.P., Goodwin J.E. (2023). Loss of endothelial glucocorticoid receptor accelerates organ fibrosis in *db/db* mice. Am. J. Physiol. Renal. Physiol..

[17] Li Y., Liu Y., Liu S., Gao M., Wang W., Chen K., Huang L., Liu Y. (2023). Diabetic vascular diseases: Molecular mechanisms and therapeutic strategies. Signal Transduct. Target. Ther..

[18] Yu M.G., Gordin D., Fu J., Park K., Li Q., King G.L. (2023). Protective factors and the pathogenesis of complications in diabetes. Endocr. Rev..

[19] Yan Z., Cai M., Han X., Chen Q., Lu H. (2023). The interaction between age and risk factors for diabetes and prediabetes: A community-based cross-sectional study. Diabetes Metab. Syndr. Obes..

[20] Banday M.Z., Sameer A.S., Nissar S. (2020). Pathophysiology of diabetes: An overview. Avicenna J. Med..

[21] Lu Y., Wang W., Liu J., Xie M., Liu Q., Li S. (2023). Vascular complications of diabetes: A narrative review. Medicine.

[22] Wilson V. (2023). An overview of complications associated with type 1 and type 2 diabetes. Nurs. Stand..

[23] Taylor S.I., Yazdi Z.S., Beitelshees A.L. (2021). Pharmacological treatment of hyperglycemia in type 2 diabetes. J. Clin. Investig..

[24] ElSayed N.A., Aleppo G., Aroda V.R., Bannuru R.R., Brown F.M., Bruemmer D., Collins B.S., Hilliard M.E., Isaacs D., Johnson E.L. (2023). 9. Pharmacologic approaches to glycemic treatment: Standards of care in diabetes—2023. Diabetes Care.

[25] Scheen A.J. (2023). Clinical pharmacology of antidiabetic drugs: What can be expected of their use?. Presse Med..

[26] DeMarsilis A., Reddy N., Boutari C., Filippaios A., Sternthal E., Katsiki N., Mantzoros C. (2022). Pharmacotherapy of type 2 diabetes: An update and future directions. Metabolism.

[27] Aronne L.J., Sattar N., Horn D.B., Bays H.E., Wharton S., Lin W.-Y., Ahmad N.N., Zhang S., Liao R., Bunck M.C. (2024). Continued treatment with tirzepatide for maintenance of weight reduction in adults with obesity: The SURMOUNT-4 randomized clinical trial. JAMA.

[28] Lisco G., Disoteo O.E., De Geronimo V., De Tullio A., Giagulli V.A., Guastamacchia E., De Pergola G., Jirillo E., Triggiani V. (2024). Is tirzepatide the new game changer in type 2 diabetes?. Endocrines.

[29] Ma J., Liu M., Wang R., Du L., Ji L. (2024). Efficacy and safety of tirzepatide in people with type 2 diabetes by baseline body mass index: An exploratory subgroup analysis of SURPASS-AP-Combo. Diabetes Obes. Metab..

[30] Sanyal A.J., Kaplan L.M., Frias J.P., Brouwers B., Wu Q., Thomas M.K., Harris C., Schloot N.C., Du Y., Mather K.J. (2024). Triple hormone receptor agonist retatrutide for metabolic dysfunction-associated steatotic liver disease: A randomized phase 2a trial. Nat. Med..

[31] Giri B., Dey S., Das T., Sarkar M., Banerjee J., Dash S.K. (2018). Chronic hyperglycemia mediated physiological alteration and metabolic distortion leads to organ dysfunction, infection, cancer progression and other pathophysiological consequences: An update on glucose toxicity. Biomed. Pharmacother..

[32] Hu Q., Chen Y., Deng X., Li Y., Ma X., Zeng J., Zhao Y. (2023). Diabetic nephropathy: Focusing on pathological signals, clinical treatment, and dietary regulation. Biomed. Pharmacother..

[33] Kim Y.H., Saha M.K., Hu Y., Kumar S., Poulton C.J., Hogan S.L., Nachman P., Jennette J.C., Nast C.C., Mottl A.K. (2023). Impact of diabetic lesions on pathology, treatment, and outcomes of glomerular diseases. Kidney.

[34] Patel S., Srivastava S., Singh M.R., Singh D. (2019). Mechanistic insight into diabetic wounds: Pathogenesis, molecular targets and treatment strategies to pace wound healing. Biomed. Pharmacother..

[35] Kim K.-S., Lee J.-S., Park J.-H., Lee E.-Y., Moon J.-S., Lee S.-K., Lee J.-S., Kim J.-H., Kim H.-S. (2021). Identification of novel biomarker for early detection of diabetic nephropathy. Biomedicines.

[36] Saleh S., Hanna G., El-Nabi S.H., El-Domiaty H., Shabaan A., Ewida S.F. (2020). Dapagliflozin, a sodium glucose cotransporter 2 inhibitors, protects cardiovascular function in type-2 diabetic murine model. J. Genet..

[37] Huo J.-L., Feng Q., Pan S., Fu W.-J., Liu Z., Liu Z. (2023). Diabetic cardiomyopathy: Early diagnostic biomarkers, pathogenetic mechanisms, and therapeutic interventions. Cell Death Discov..

[38] Cusi K. (2024). Nonalcoholic fatty liver disease in diabetes: A call to action. Diabetes Spectr..

[39] Dutta B.J., Singh S., Seksaria S., Das Gupta G., Singh A. (2022). Inside the diabetic brain: Insulin resistance and molecular mechanism associated with cognitive impairment and its possible therapeutic strategies. Pharmacol. Res..

[40] Sugandh F., Chandio M., Raveena F., Kumar L., Karishma F., Khuwaja S., Memon U.A., Bai K., Kashif M., Varrassi G. (2023). Advances in the management of diabetes mellitus: A focus on personalized medicine. Cureus.

[41] Kannan S., Chellappan D.K., Kow C.S., Ramachandram D.S., Pandey M., Mayuren J., Dua K., Candasamy M. (2023). Transform diabetes care with precision medicine. Health Sci. Rep..

[42] Raj G.M., Mathaiyan J. (2021). Precision medicine in diabetes-Finally some light at the end of the tunnel?. Br. J. Clin. Pharmacol..

[43] Ahmad E., Sargeant J.A., Zaccardi F., Khunti K., Webb D.R., Davies M.J. (2020). Where does metformin stand in modern day management of type 2 diabetes?. Pharmaceuticals.

[44] Bailey C.J. (2024). Metformin: Therapeutic profile in the treatment of type 2 diabetes. Diabetes Obes. Metab..

[45] Zhu H., Jia Z., Li Y.R., Danelisen I. (2023). Molecular mechanisms of action of metformin: Latest advances and therapeutic implications. Clin. Exp. Med..

[46] Lewis A., Williams K., Oroszi T. (2024). Metformin—Pharmacokinetic and Pharmacodynamics Journey Through the Body. Pharmacol. Pharm..

[47] Cheng M., Ren L., Jia X., Wang J., Cong B. (2024). Understanding the action mechanisms of metformin in the gastrointestinal tract. Front. Pharmacol..

[48] Buczyńska A., Sidorkiewicz I., Krętowski A.J., Adamska A. (2024). Examining the clinical relevance of metformin as an antioxidant intervention. Front. Pharmacol..

[49] Wang Y.-W., He S.-J., Feng X., Cheng J., Luo Y.-T., Tian L., Huang Q. (2017). Metformin: A review of its potential indications. Drug Des. Dev. Ther..

[50] Corcoran C., Jacobs T.F. (2025). Metformin. StatPearls [Internet].

[51] Andraos J., Smith S.R., Tran A., Pham D.Q. (2024). Narrative review of data supporting alternate first-line therapies over metformin in type 2 diabetes. J. Diabetes Metab. Disord..

[52] Han J., Li Y., Liu X., Zhou T., Sun H., Edwards P., Gao H., Yu F.-S., Qiao X. (2018). Metformin suppresses retinal angiogenesis and inflammation in vitro and in vivo. PLoS ONE.

[53] Kim Y.S., Kim M., Choi M.Y., Lee D.H., Roh G.S., Kim H.J., Kang S.S., Cho G.J., Kim S.-J., Yoo J.-M. (2017). Metformin protects against retinal cell death in diabetic mice. Biochem. Biophys. Res. Commun..

[54] Nahar N., Mohamed S., Mustapha N.M., Lau S., Ishak N.I.M., Umran N.S. (2021). Metformin attenuated histopathological ocular deteriorations in a streptozotocin-induced hyperglycemic rat model. Naunyn-Schmiedeberg’s Arch. Pharmacol..

[55] Oubaha M., Miloudi K., Dejda A., Guber V., Mawambo G., Germain M.-A., Bourdel G., Popovic N., Rezende F.A., Kaufman R.J. (2016). Senescence-associated secretory phenotype contributes to pathological angiogenesis in retinopathy. Sci. Transl. Med..

[56] Xu L., Kong L., Wang J., Ash J.D. (2018). Stimulation of AMPK prevents degeneration of photoreceptors and the retinal pigment epithelium. Proc. Natl. Acad. Sci. USA.

[57] Qu S., Zhang C., Liu D., Wu J., Tian H., Lu L., Xu G.-T., Liu F., Zhang J. (2020). Metformin protects ARPE-19 cells from glyoxal-induced oxidative stress. Oxid. Med. Cell. Longev..

[58] Zhao X., Liu L., Jiang Y., Silva M., Zhen X., Zheng W. (2020). Protective effect of metformin against hydrogen peroxide-induced oxidative damage in human retinal pigment epithelial (RPE) cells by enhancing autophagy through activation of AMPK pathway. Oxidative Med. Cell. Longev..

[59] Yi Q.-Y., Deng G., Chen N., Bai Z.-S., Yuan J.-S., Wu G.-H., Wang Y.-W., Wu S.-J. (2016). Metformin inhibits development of diabetic retinopathy through inducing alternative splicing of VEGF-A. Am. J. Transl. Res..

[60] Zhang Y., Chen F., Wang L. (2017). Metformin inhibits development of diabetic retinopathy through microRNA-497a-5p. Am. J. Transl. Res..

[61] Luodan A., Zou T., He J., Chen X., Sun D., Fan X., Xu H. (2019). Rescue of retinal degeneration in rd1 mice by intravitreally injected metformin. Front. Mol. Neurosci..

[62] Ying Y., Ueta T., Jiang S., Lin H., Wang Y., Vavvas D., Wen R., Chen Y.-G., Luo Z. (2017). Metformin inhibits ALK1-mediated angiogenesis via activation of AMPK. Oncotarget.

[63] Zhang J.Y., Xiao J., Xie B., Barba H., Boachie-Mensah M., Shah R.N., Nadeem U., Spedale M., Dylla N., Lin H. (2023). Oral metformin inhibits choroidal neovascularization by modulating the gut-retina axis. Investig. Opthalmol. Vis. Sci..

[64] Xiao J.F., Luo W., Mani A., Barba H., Solanki A., Droho S., Lavine J.A., Skondra D. (2024). Intravitreal Metformin Protects Against Choroidal Neovascularization and Light-Induced Retinal Degeneration. Int. J. Mol. Sci..

[65] Wang X., Liang X., Huang S., Wei M., Xu Y., Chen X., Miao Y., Zong R., Lin X., Li S. (2025). Metformin inhibits pathological retinal neovascularization but promotes retinal fibrosis in experimental neovascular age-related macular degeneration. Front. Pharmacol..

[66] Hasan I., Rashid T., Jaikaransingh V., Heilig C., Abdel-Rahman E.M., Awad A.S. (2024). SGLT2 inhibitors: Beyond glycemic control. J. Clin. Transl. Endocrinol..

[67] Yankah R.K., Anku E.K., Eligar V. (2024). Sodium-glucose cotransporter-2 inhibitors and cardiovascular protection among patients with type 2 diabetes mellitus: A systematic review. J. Diabetes Res..

[68] Seidu S., Alabraba V., Davies S., Newland-Jones P., Fernando K., Bain S.C., Diggle J., Evans M., James J., Kanumilli N. (2024). SGLT2 Inhibitors—The new standard of care for cardiovascular, renal and metabolic protection in type 2 diabetes: A narrative review. Diabetes Ther..

[69] Ciardullo S., Morieri M.L., Daniele G., Fiorentino T.V., Mezza T., Tricò D., Consoli A., Del Prato S., Giorgino F., Piro S. (2024). GLP1-GIP receptor co-agonists: A promising evolution in the treatment of type 2 diabetes. Acta Diabetol..

[70] Pescariu S.A., Elagez A., Nallapati B., Bratosin F., Bucur A., Negru A., Gaita L., Citu I.M., Popa Z.L., Barata P.I. (2024). Examining the impact of ertugliflozin on cardiovascular outcomes in patients with diabetes and metabolic syndrome: A systematic review of clinical trials. Pharmaceuticals.

[71] Cheng Q., Zou S., Feng C., Xu C., Zhao Y., Shi X., Sun M. (2023). Effect of ertugliflozin on renal function and cardiovascular outcomes in patients with type 2 diabetes mellitus: A systematic review and meta-analysis. Medicine.

[72] Collins L., Costello R.A. (2025). Glucagon-like peptide-1 receptor agonists. StatPearls [Internet].

[73] Mann J.F.E., Rossing P., Bakris G., Belmar N., Bosch-Traberg H., Busch R., Charytan D.M., Hadjadj S., Gillard P., Górriz J.L. (2024). Effects of semaglutide with and without concomitant SGLT2 inhibitor use in participants with type 2 diabetes and chronic kidney disease in the FLOW trial. Nat. Med..

[74] Zheng Z., Zong Y., Ma Y., Tian Y., Pang Y., Zhang C., Gao J. (2024). Glucagon-like peptide-1 receptor: Mechanisms and advances in therapy. Signal Transduct. Target. Ther..

[75] Ilias I., Zabuliene L., Rizzo M. (2025). GLP-1 receptor agonists in diabetes and weight loss: The double-edged sword of innovation and risks. Front. Clin. Diabetes Healthc..

[76] Cai W., Zhang R., Yao Y., Wu Q., Zhang J. (2024). Tirzepatide as a novel effective and safe strategy for treating obesity: A systematic review and meta-analysis of randomized controlled trials. Front. Public Health.

[77] Mahar M.U., Mahmud O., Ahmed S., Qureshi S.A., Kakar W.G., Fatima S.S. (2024). The effects of tirzepatide on lipid profile: A systematic review and meta-analysis of randomized controlled trials. J. Obes. Metab. Syndr..

[78] France N.L., Syed Y.Y. (2024). Tirzepatide: A review in type 2 diabetes. Drugs.

[79] Fontanella R.A., Ghosh P., Pesapane A., Taktaz F., Puocci A., Franzese M., Feliciano M.F., Tortorella G., Scisciola L., Sommella E. (2024). Tirzepatide prevents neurodegeneration through multiple molecular pathways. J. Transl. Med..

[80] Almuttairi R.S. (2023). The effects of metformin treatment on diabetic albino rats’ pancreas, liver, and kidney histology. Arch. Razi Inst..

[81] Salem A.A.M. (2022). Biochemical and histopathological evaluation of the effect of metformin and metformin nanoparticles against alloxan-induced diabetes in Rats. Benha Veter. Med. J..

[82] Mobasher M.A., El-Tantawi H.G., El-Said K.S. (2020). Metformin ameliorates oxidative stress induced by diabetes mellitus and hepatocellular carcinoma in rats. Rep. Biochem. Mol. Biol..

[83] Dallak M., Bin-Jaliah I., Al-Hashem F., Kamar S.S., Abdel Kader D.H., Amin S.N., Haidara M.A., Al-Ani B. (2018). Metformin pretreatment ameliorates diabetic nephropathy induced by a combination of high fat diet and streptozotocin in rats. Int. J. Morphol..

[84] Zhang S., Xu H., Yu X., Wu Y., Sui D. (2017). Metformin ameliorates diabetic nephropathy in a rat model of low-dose streptozotocin-induced diabetes. Exp. Ther. Med..

[85] Dawood A.F., Alzamil N.M., Hewett P.W., Momenah M.A., Dallak M., Kamar S.S., Kader D.H.A., Yassin H., Haidara M.A., Maarouf A. (2022). Metformin protects against diabetic cardiomyopathy: An association between desmin-sarcomere injury and the iNOS/mTOR/TIMP-1 fibrosis axis. Biomedicines.

[86] Yang Z., Wang M., Zhang Y., Cai F., Jiang B., Zha W., Yu W. (2020). Metformin ameliorates diabetic cardiomyopathy by activating the PK2/PKR pathway. Front. Physiol..

[87] Niu C., Chen Z., Kim K.T., Sun J., Xue M., Chen G., Li S., Shen Y., Zhu Z., Wang X. (2019). Metformin alleviates hyperglycemia-induced endothelial impairment by downregulating autophagy via the Hedgehog pathway. Autophagy.

[88] Yan Y., Li T., Li Z., He M., Wang D., Xu Y., Yang X., Bai Y., Lao Y., Zhang Z. (2021). Metformin suppresses the progress of diabetes-accelerated atherosclerosis by inhibition of vascular smooth muscle cell migration through AMPK-Pdlim5 pathway. Front. Cardiovasc. Med..

[89] Naito T., Yamanaka Y., Tokuda K., Sato N., Tajima T., Tsukamoto M., Suzuki H., Kawasaki M., Nakamura E., Sakai A. (2024). Effects of metformin on knee joint capsule fibrosis in a diabetic mouse model. Bone Jt. Res..

[90] Han J.-X., Luo L.-L., Wang Y.-C., Miyagishi M., Kasim V., Wu S.-R. (2023). SGLT2 inhibitor empagliflozin promotes revascularization in diabetic mouse hindlimb ischemia by inhibiting ferroptosis. Acta Pharmacol. Sin..

[91] Matthews J.R., Schlaich M.P., Rakoczy E.P., Matthews V.B., Herat L.Y. (2022). The effect of SGLT2 inhibition on diabetic kidney disease in a model of diabetic retinopathy. Biomedicines.

[92] Farias R.S., Silva-Aguiar R.P., Teixeira D.E., Gomes C.P., Pinheiro A.A.S., Peruchetti D.B., Caruso-Neves C. (2023). Inhibition of SGLT2 co-transporter by dapagliflozin ameliorates tubular proteinuria and tubule-interstitial injury at the early stage of diabetic kidney disease. Eur. J. Pharmacol..

[93] Dia B., Alkhansa S., Njeim R., Al Moussawi S., Farhat T., Haddad A., Riachi M.E., Nawfal R., Azar W.S., Eid A.A. (2023). SGLT2 inhibitor-dapagliflozin attenuates diabetes-induced renal injury by regulating inflammation through a CYP4A/20-HETE signaling mechanism. Pharmaceutics.

[94] Kim H.K., Kim Y., Kim R.-H., Lee S., Yang Y., Park H., Jeon N., Lee M., Lee Y.-H., Cha B.-S. (2022). Effects of ertugliflozin on podocyte in high-fat diet–induced diabetic kidney disease model, in vivo and in vitro. Diabetes.

[95] Liu P., Zhang Z., Wang J., Zhang X., Yu X., Li Y. (2021). Empagliflozin protects diabetic pancreatic tissue from damage by inhibiting the activation of the NLRP3/caspase-1/GSDMD pathway in pancreatic β cells: In vitro and in vivo studies. Bioengineered.

[96] Croteau D., Luptak I., Chambers J.M., Hobai I., Panagia M., Pimentel D.R., Siwik D.A., Qin F., Colucci W.S. (2021). Effects of sodium-glucose linked transporter 2 inhibition with ertugliflozin on mitochondrial function, energetics, and metabolic gene expression in the presence and absence of diabetes mellitus in mice. J. Am. Heart Assoc..

[97] Zhao Q., Dong J., Liu H., Chen H., Yu H., Ye S., Yu S., Li Y., Qiu L., Song N. (2024). Design and discovery of a highly potent ultralong-acting GLP-1 and glucagon co-agonist for attenuating renal fibrosis. Acta Pharm. Sin. B.

[98] Iwai S., Kaji K., Nishimura N., Kubo T., Tomooka F., Shibamoto A., Suzuki J., Tsuji Y., Fujinaga Y., Kitagawa K. (2023). Glucagon-like peptide-1 receptor agonist, semaglutide attenuates chronic liver disease-induced skeletal muscle atrophy in diabetic mice. Biochim. Biophys. Acta Mol. Basis Dis..

[99] Soto-Catalán M., Opazo-Ríos L., Quiceno H., Lázaro I., Moreno J.A., Gómez-Guerrero C., Egido J., Mas-Fontao S. (2024). Semaglutide improves liver steatosis and de novo lipogenesis markers in obese and type-2-diabetic mice with metabolic-dysfunction-associated steatotic liver disease. Int. J. Mol. Sci..

[100] Li R., Ye Z., She D., Fang P., Zong G., Hu K., Kong D., Xu W., Li L., Zhou Y. (2022). Semaglutide may alleviate hepatic steatosis in T2DM combined with NFALD mice via miR-5120/ABHD6. Drug Des. Dev. Ther..

[101] Schwasinger-Schmidt T., Robbins D.C., Williams S.J., Novikova L., Stehno-Bittel L. (2013). Long-term liraglutide treatment is associated with increased insulin content and secretion in β-cells, and a loss of α-cells in ZDF rats. Pharmacol. Res..

[102] Chen L.-N., Lyu J., Yang X.-F., Ji W.-J., Yuan B.-X., Chen M.-X., Ma X., Wang B. (2013). Liraglutide ameliorates glycometabolism and insulin resistance through the upregulation of GLUT4 in diabetic KKAy mice. Int. J. Mol. Med..

[103] Alobaid S.M., Alshahrani R.M., Alonazi A.S., Alrasheed N.M., Alamin M.A., Alshammari T.K., Bin Dayel A.F., Elnagar D.M., Alotaibi R.R., Almuthnabi L.A. (2024). Liraglutide attenuates diabetic cardiomyopathy via the ILK/PI3K/AKT/PTEN signaling pathway in rats with streptozotocin-induced type 2 diabetes mellitus. Pharmaceuticals.

[104] Martos-Guillami N., Vergara A., Llorens-Cebrià C., Motto A.E., Martínez-Díaz I., Gonçalves F., Garcias-Ramis M.M., Allo-Urzainqui E., Narváez A., Bermejo S. (2024). SGLT2i and GLP1-RA exert additive cardiorenal protection with a RAS blocker in uninephrectomized db/db mice. Front. Pharmacol..

[105] Tian Y., Tian R., Juan H., Guo Y., Yan P., Cheng Y., Li R., Wang B. (2025). GLP-1/GIP dual agonist tirzepatide normalizes diabetic nephropathy via PI3K/AKT mediated suppression of oxidative stress. Int. Immunopharmacol..

[106] Iwamoto Y., Kimura T., Dan K., Iwamoto H., Sanada J., Fushimi Y., Katakura Y., Shimoda M., Yamasaki Y., Nogami Y. (2024). Tirzepatide, a dual glucose-dependent insulinotropic polypeptide/glucagon-like peptide 1 receptor agonist, exhibits favourable effects on pancreatic β-cells and hepatic steatosis in obese type 2 diabetic db/db mice. Diabetes Obes. Metab..

[107] Yuan J., Liu W., Jiang X., Huang Y., Zong L., Ding H., Shen X., Sun Y., Feng X., Li X. (2024). Molecular dynamics-guided optimization of BGM0504 enhances dual-target agonism for combating diabetes and obesity. Sci. Rep..

[108] Jeong B.-K., Choi W.-I., Choi W., Moon J., Lee W.H., Choi C., Choi I.Y., Lee S.-H., Kim J.K., Ju Y.S. (2024). A male mouse model for metabolic dysfunction-associated steatotic liver disease and hepatocellular carcinoma. Nat. Commun..

[109] Yang Y., Wang Y., Zhou Y., Deng J., Wu L. (2025). Tirzepatide alleviates oxidative stress and inflammation in diabetic nephropathy via IL-17 signaling pathway. Mol. Cell. Biochem..

